# Increased Feeding and Food Hoarding following Food Deprivation Are Associated with Activation of Dopamine and Orexin Neurons in Male Brandt's Voles

**DOI:** 10.1371/journal.pone.0026408

**Published:** 2011-10-27

**Authors:** Xue-Ying Zhang, Hui-Di Yang, Qiang Zhang, Zuoxin Wang, De-Hua Wang

**Affiliations:** 1 State Key Laboratory of Integrated Management of Pest Insects and Rodents, Institute of Zoology, Chinese Academy of Sciences, Beijing, China; 2 Department of Psychology and Program in Neuroscience, Florida State University, Tallahassee, Florida, United States of America; University of Chicago, United States of America

## Abstract

Small mammals usually face energetic challenges, such as food shortage, in the field. They have thus evolved species-specific adaptive strategies for survival and reproductive success. In the present study, we examined male Brandt's voles (*Lasiopodomys brandtii*) for their physiological, behavioral, and neuronal responses to food deprivation (FD) and subsequent re-feeding. Although 48 hr FD induced a decrease in body weight and the resting metabolic rate (RMR), such decreases did not reach statistical significance when compared to the control males that did not experience FD. During the first 2 hr of re-feeding following 48 hr FD, voles showed higher levels of feeding than controls. However, when permitted to hoard food, FD voles showed an increase in food hoarding, rather than feeding, compared to the controls. Further, both feeding and food hoarding induced an increase in neuronal activation, measured by Fos-ir, in a large number of brain areas examined. Interestingly, feeding and food hoarding also induced an increase in the percentage of tyrosine hydroxylase immunoreactive (TH-ir) cells that co-expressed Fos-ir in the ventral tegmental area (VTA), whereas both FD and feeding induced an increase in the percentage of orexin-ir cells that co-expressed Fos-ir in the lateral hypothalamus (LH). Food hoarding also increased orexin-ir/Fos-ir labeling in the LH. Together, our data indicate that food-deprived male Brandt's voles display enhanced feeding or food hoarding dependent upon an environmental setting. In addition, changes in central dopamine and orexin activities in selected brain areas are associated with feeding and hoarding behaviors following FD and subsequent re-feeding.

## Introduction

Food availability is an important environmental cue that affects animal fitness, such as body weight, development, survival, and reproduction [1]. When faced with limited food availability, many small mammals, such as rats, mice, voles, and gerbils, with relatively high metabolic rates can engage in several behaviors to lower energy expenditure. They can decrease resting metabolic rate (RMR) and non-shivering thermogenesis (NST) [2]–[4], reduce physical activity [5], or even enter torpor [6] to conserve energy. Nevertheless, they still show decreases in body weight and fat following food shortage [3], [7].

Many rodent species regain body weight following food shortage-induced weight loss by increasing feeding (food intake) [8]. In contrast, other species, such as Siberian hamsters (*Phodopus sungorus*) and Syrian hamsters (*Mesocricetus auratus*), were reported to increase hoarding rather than feeding when given access to food following food shortage [9]–[10]. These compensatory increases in feeding or food hoarding to restore energy balance may be a “hard-wired”, evolutionarily conserved mechanism to ensure animal's survival and reproductive fitness [1]. However, we still know very little about the neuromechanisms underlying these behaviors.

The mediobasal hypothalamus is considered to be the main integrator of peripheral hormonal signals to control homeostatic body regulation [11]–[13]. One of the most studied hormonal signals, leptin, is produced by adipose tissue. Serum leptin levels fall with fat mass loss in response to starvation [14]–[15], triggering a powerful activation of orexigenic neurons [16]–[17] and suppression of anorexigenic neurons in the arcuate nucleus (ARC) [18]. Interestingly, a recent study showed that ARC destruction did not block food deprivation-induced increases in food foraging and hoarding [19], indicating that distinct brain regions other than the ARC may be involved in feeding and hoarding regulation. The orexin/hypocretin system in the lateral hypothalamus (LH) is suggested to interact with brain dopamine systems and to participate in a variety of behavioral and physiological processes associated with both appetitive and aversive (e.g. stress/fear) conditions [20]–[21].

Brandt's voles (*Lasiopodomys brandtii*) mainly inhabit in the grasslands of Inner Mongolia of China, Mongolia, and the region of Beigaer in Russia. They are non-hibernating rodents and hoard food in the fall for the long winter. When exposed to environmental stressors, such as food deprivation or cold temperature, the voles were found to engage in several adaptive behaviors, such as increased feeding, to regulate energy balance and to ensure their survival [14], [22]–[23]. Based on these findings, we hypothesized that feeding and food hoarding are mediated by a neurocircuit involving coordination of multiple brain areas and neurochemicals. In the present study using c-Fos (the protein product of the immediate-early gene *c-fos*) immunohistochemistry, we examined the patterns of neuronal activation in response to food deprivation and subsequent re-feeding in male Brandt's voles. We also investigated whether the mesocorticolimbic dopamine system and lateral hypothalamic orexin system were activated during this process. Our overarching hypothesis was that the activation and interaction of central dopamine and orexin systems are associated with feeding and food hoarding during energetic challenges in Brandt's voles.

## Materials and Methods

### Ethics Statement

All experimental protocols were approved by the Committee on the Ethics of Animal and Medicine of the Institute of Zoology, Chinese Academy of Sciences (Permit Number: IOZ11012). All researchers and students had received appropriate training and certified before performing animal studies.

### Subjects

Subjects were sexually naive adult male Brandt's voles that were offspring of a laboratory breeding colony started with field-captured animals in May 1999. After weaning at 21 days of age, voles were housed as same sex sibling pairs in plastic cages (30×15×20 cm) and maintained in temperature- (23±1°C) and humidity-controlled rooms under a 16L∶8D photoperiod with lights on at 0400. All animals were provided with standard rabbit pellet chow (KeAo Feed Co., Beijing) and water *ad libitum*. At 90–120 days of age, subjects were housed individually for 2 weeks prior to being assigned into experimental groups.

### Experimental Procedures

Resting metabolic rate (RMR) measurement: Using our established methods [24], RMR was measured by an open-circuit respirometer (FoxBox, Sable Systems International Inc., Las Vegas, NV, USA) between 0800 and 1700. RMR was assessed from the rate of O_2_ consumption and CO_2_ production at 30°C (within Brandt's vole's thermal neutral zone) (constant-temperature incubator; model LRH-250; Yiheng Co., Shanghai, CHN). The subject was placed in a LOCK and LOCK stackable airtight container (200×130×85 mm, volume 1.4 L; HPL817H; LOCK and LOCK Co., KR) for 2·hr. The flow rate of incurrent and excurrent air (dried with anhydrous CaSO_4_; W. A. Hammond Drierite Co., USA) was approximately 400–600·ml/min and 100·ml/min, respectively. The basal levels of oxygen and carbon dioxide concentrations were measured before and after each test. Oxygen consumption was recorded at 10-s intervals. Each subject was in the metabolic chamber for at least 2 hr. The rate of oxygen consumption was calculated by the following equation. RMR was estimated from the stable lowest consecutive rate of oxygen consumption over 5·min.
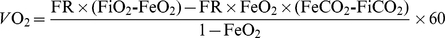

*V*O_2_ = oxygen consumption (mlO_2_/h), FR = flow rate (ml/min), Fi = input fractional concentration (%), Fe = excurrent fractional concentration (%).

Food hoarding: The food hoarding apparatus was designed according to previous studies [25]–[26], and was used in our recent study [27]. The subject was transferred to a 32×21×16 cm clear plastic cage, referred to as the “home cage”, where water and four cotton balls were provided to facilitate nesting. Food pellets were provided on the lid of another clear plastic box (30×15×20 cm), named the “food cage”. Each home cage was connected to a food cage by a 90 cm long piece of translucent plastic tubing (5 cm in diameter). No subjects were observed to nap in the food cage, to move nesting material to the food cage, or to move food from the home cage back to food cage during food hoarding. All voles visited the food cage. Before each hoarding session, the amount of food offered was weighed and then put directly into the food cage. Both the amount of food that remained in the food cage and the amount that was hoarded by the subject to the home cage were weighed at the end of each session. The food intake was calculated by subtracting the amount of food that remained and was hoarded from that offered.

Tissue preparation: Subjects were anesthetized with an overdose of pentobarbital (50 mg/kg) injected intraperitoneally. Subjects were then transcardially perfused with saline followed by ice-cold 4% paraformaldehyde in 0.1 M phosphate buffered saline (PBS, pH 7.4). Brains were immediately removed, postfixed for 2 hr in the same fixative at 4°C, and infiltrated with 30% sucrose in 0.1 M PBS until they sank at 4°C. Serial coronal sections in 40 µm thickness were cut on a cryostat (Leica CM1950; Leica, Wetzlar, Germany) and stored in PBS with 1% sodium azide until being processed for immunoreactive staining for c-Fos, tyrosine hydroxylase (TH; a rate limit enzyme for dopamine conversion), and orexin.

Immunoreactive staining for Fos, TH, and orexin: A set of floating brain sections at 160 µm intervals was processed for c-Fos immunocytochemistry, as described in our previous studies [27]–[28]. Briefly, sections were incubated in 3% hydrogen peroxide in 0.1 M PBS for 30 min; rinsed in 0.1 M PBS three times; incubated in 0.5% Triton X-100 in 0.1 M PBS with 10% normal goat serum for 1 h at room temperature, and then in rabbit anti-Fos polyclonal IgG (c-Fos [4]-G: *sc*-52; 1∶30,000; Santa Cruz Biotechnology, Santa Cruz, CA) in 0.5% Triton X-100 in 0.1 M PBS with 2% normal goat serum at 4°C overnight. Sections were rinsed in 0.5% Triton X-100 in 0.1 M PBS for 3 times and incubated in biotinylated goat anti-rabbit IgG (BA-1000; 1∶500; Vector, Burlingame, CA) in 0.5% Triton X-100 in 0.1 M PBS with 2% normal goat serum for 2 hr. Sections were rinsed in 0.1 M PBS, incubated in avidin-biotin complex (Vectastain Elite, Vector, Burlingame, CA) in 0.1 M PBS for 90 min, and rinsed in 0.1 M PBS. Nuclear labeling was immunoreactively stained by using 3′-diaminobenzidine (DAB/H_2_O_2_ tablet; Sigma, St. Louis, MO) and 8 mg of NiCl powder deionized/distilled H_2_O for 15 min, revealing black punctate nuclear staining. Sections were then rinsed in 0.1 M PBS.

Next, sections were incubated in 0.1% Triton X-100 in 0.1 M PBS with 5% normal goat serum for 30 min and then incubated in rabbit anti-TH (AB152; 1∶8,000; Chemicon, Temecula, CA) in 0.1% Triton X-100 in 0.1 M PBS with 2% normal goat serum overnight. Sections were rinsed for 20 min, and incubated in biotinylated goat anti-rabbit IgG (1∶500) for 2 hr at room temperature. Sections were rinsed and incubated in avidin-biotin complex (Vector) in 0.1 M PBS for 90 min, and rinsed in 0.1 M PBS. Cytoplasmic labeling was immunoreactively stained by using a Vector DAB Kit in 5 ml ddH_2_O for 3 min, revealing brown cytoplasmic staining. Sections were rinsed in 0.1 M PBS.

Another set of brain sections at 160 µm intervals was processed for Fos/orexin double immunocytochemistry. The same protocol outlined above was used except that the primary antibody for orexin labeling was goat anti-orexin (sc-8070; 1∶2000; Santa Cruz Biotechnology) and the secondary antibody was donkey anti-goat IgG (705-066-147; 1∶500; Jackson ImmunoResearch, West Grove, PA).

The specificity of antibodies was assessed in rats and voles previously [28]–[29], and also corroborated with control sections that were stained with preimmune rabbit (for c-Fos and TH) or goat (for orexin) serum; and control sections in which either the primary antibody, secondary antibody or the ABC complex was omitted.

### Data Quantification and Analysis

Photomicrographs were captured by using a Nikon eclipse 80i microscope with a SPOT RT_KE_ 7.4 Slider (Diagnostic Instruments) camera and SPOT advanced (version 4.0.9) software. All cell counts were performed blind to treatment. Profile cell counting for all stained cells per brain area was used to examine neurons containing Fos-ir, TH-ir, orexin-ir, Fos-ir/TH-ir, or Fos-ir/orexin-ir, respectively. Stained cells were identified and quantified bilaterally in the following brain regions: the nucleus accumbens (NAcc); lateral septum (LS); bed nucleus of the stria terminalis (BNST); medial preoptic area (MPOA); suprachiasmatic nucleus (SCN); anterior (AH), lateral (LH), paraventricular (PVN), dorsomedial (DMH), and arcuate (ARC) nuclei of the hypothalamus; paraventricular nucleus of the thalamus (PVT); medial (MeA), anterior cortical (ACo) and central (CeA) nuclei of the amygdala; and ventral tegmental area (VTA). At least 3 sections were counted bilaterally for each brain region. The image for Fos staining was taken under 100× magnification, while double labeling was taken under 200× and 400× magnification. Brain regions were identified and defined using the rat brain atlas [30].

All data were analyzed using SPSS 13.0 software package. Prior to the statistical analyses, data were examined for normality and homogeneity of variance, using Kolmogorov–Smirnov and Levene tests, respectively. Differences in body weight, food intake, RMR, and food hoarding during the experimental course were analyzed by a repeated measures one-way analysis of variance (ANOVA) followed by least-significant difference (LSD) posthoc tests for three or more groups, or by independent-samples *t* tests for comparisons between two groups. Group differences in neurons stained for Fos-ir, TH-ir, orexin-ir, Fos-ir/TH-ir, and Fos-ir/orexin-ir were analyzed by a one-way ANOVA followed by LSD posthoc tests for feeding condition (3 groups), or by independent-samples *t* tests for hoarding condition (2 groups). Results are presented as percent change over the mean of the baseline control ± SEM, and *p*≤0.05 was considered to be statistically significant.

### Experimental Design

Experiment 1 was designed to test the metabolic response to food deprivation and subsequent re-feeding.

**Figure 1:**
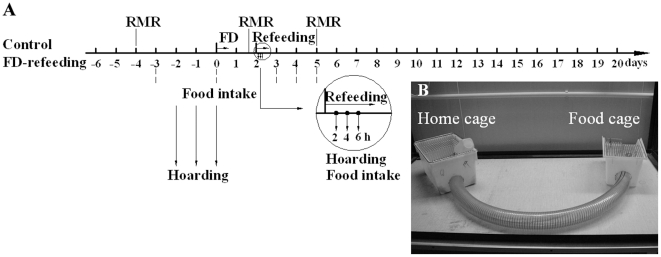
Experimental paradigm and food hoarding apparatus. Experimental paradigm (panel A) illustrating a time line by which food intake, resting metabolic rate (RMR), and food hoarding during re-feeding following 48 hr food deprivation (FD) were measured. The food hoarding apparatus (panel B) consisted of a home cage and a food cage connected by a plastic tube.

Fourteen weight-matched male voles were randomly assigned into one of the two treatment groups: one was given food and water *ad libitum* continuously (Control, *n* = 7) and another was food deprived (FD) (starting at 0700 for 48 hr) and then provided with food and water *ad libitum* for 18 days (FD-refeeding, *n* = 7). Subjects in the second group had free access to water during food deprivation. Body weight was measured every 2 days. RMR was measured before food deprivation (baseline), on the second day of food deprivation, and on the third day of re-feeding, respectively. Food intake was determined for 3 consecutive days before food deprivation (baseline), every 2 hr (total 6 hr) on the first day of re-feeding, and every 24 hr throughout the first, second, and third days of re-feeding. The time points to measure food intake and RMR are shown in Fig. 1A.

Experiment 2 was designed to reveal the effects of food deprivation on food hoarding behavior.

Twenty weight-matched male voles were put into the food hoarding apparatus (Fig. 1B), as described in our recent study [27], for 4 weeks to get familiar with the environment. During this period, 6 voles showed little or no food hoarding, and thus were excluded from the experiment. The remaining 14 voles were randomly assigned into one of the two experimental groups, Control (*n* = 7) and FD-rehoarding (*n* = 7), as described in Experiment 1. In both groups, food pellets were put directly into the food cage to allow animals to display food hoarding behavior. We measured subject's food hoarding for 2 hr each day for 3 d prior to food deprivation (baseline), and for 3 continuous times (total 6 hr) on the first day of re-hoarding. The time points to measure food hoarding are shown in Fig. 1A. Food intake during the hoarding session was also calculated.

Experiment 3 was designed to examine activation of dopamine and orexin neurons in the brain associated with food deprivation and subsequent re-feeding.

This was examined under two experimental conditions: food deprivation with re-feeding and food deprivation with food hoarding. In the first condition, twenty-four weight-matched male voles were randomly assigned into one of 3 experimental groups including continuous *ad libitum* feeding (Control, *n* = 7), food deprivation for 48 hr (FD, *n* = 9), and 2 hr *ad libitum* re-feeding following 48-hr FD (RF2h, *n* = 8). Food deprivation started at 0900 for the FD group and at 0700 for the RF2h group, so that all subjects were sacrificed at the same time two days later. In the second condition, subjects were permitted to hoard food, and randomly assigned into either Control (*n* = 7) or 2 hr food hoarding following 48 hr FD (RH2h, *n* = 7) groups. In both conditions, all subjects had free access to water during food deprivation. Subjects were sacrificed immediately after the behavioral test and their brain sections were processed for Fos-ir, TH-ir, and orexin-ir staining.

## Results

### Physiological responses to food-deprivation and re-feeding

**Figure 2:**
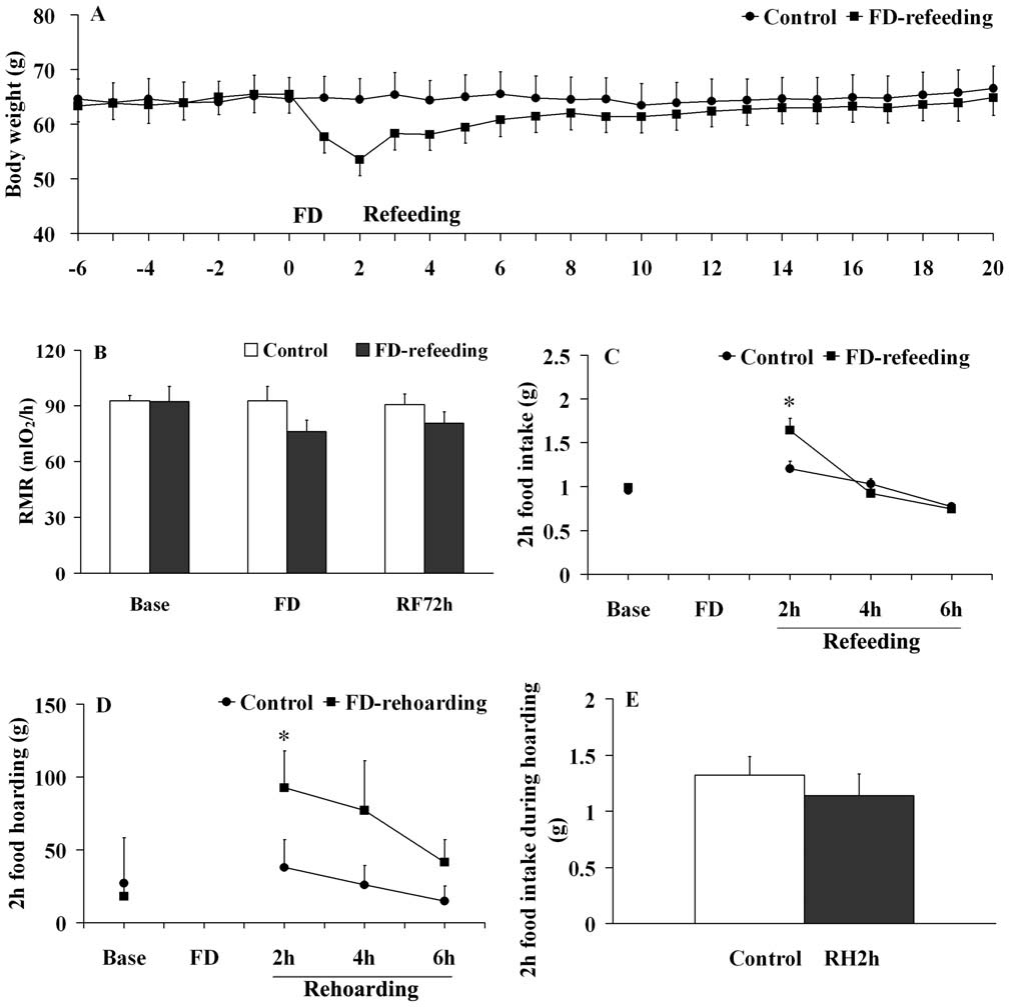
Body weight, RMR, food intake and food hoarding. Panel A showed the changes in body weight over the course of the baseline, 48 hr food deprivation (FD), and subsequent re-feeding (refeeding) period in male Brandt's voles. Panel B: No differences were found in RMR at basal level (Base), during FD, and 72 hr after re-feeding (RF72h) between intact male voles (Control) and voles that went through 48 FD followed by re-feeding (FD-refeeding). Panel C: The FD-refeeding males increased food intake by 33% within 2 hr after they were re-fed *ad libitum*, compared to the controls. Panel D: The FD-rehoarding males increased the amount of food hoarding by 140% within 2 hr after they were re-fed *ad libitum*, compared to the controls. Panel E: There was no difference in the amount of food intake during food hoarding between control voles and voles that went through 48 hr FD followed by 2 hr food hoarding (RH2h). Data are presented as mean ± SEM. * *P*<0.05.

Control and FD-refeeding males did not differ in body weight prior to the experimental manipulation (Fig. 2A). Two days of FD led to a 12%, but not significant, decrease in body weight compared to the control (*t*_12_ = 1.64, *P* = 0.12). Over the course, significant differences in body weight were found within the FD-refeeding group (*F*_26,156_ = 21.60, *P* = 0.001), but not in the control (*F*_26,156_ = 1.35, *P* = 0.14). In the FD-refeeding group, one or two days of FD resulted in significant decreases in body weight compared to the baseline levels, and body weight completely recovered within 2 weeks after refeeding.

No significant group differences in RMR were found in the basal levels, on the 2^nd^ day of FD (a 18% decrease in FD voles vs. control) or on the 3^rd^ day of re-feeding (Fig. 2B). Over the course, no significant differences were found in RMR within both the control (*F*_2,10_ = 0.15, *P* = 0.87) and FD-refeeding groups (*F*_2,10_ = 0.45, *P* = 0.65).

### Behavioral responses to food-deprivation and re-feeding

Control and RF2h males did not differ in the baseline levels of food intake prior to the experimental manipulation (Fig. 2C). However, RF2h males significantly increased food intake within 2 hr after they were re-fed *ad libitum*, compared to the controls (*t*_12_ = 2.71, *P* = 0.02; Fig. 2C). This group difference in food intake disappeared thereafter (Fig. 2C). There were significant differences in food intake during the course both in the control (*F*_3,18_ = 16.68, *P* = 0.001) and FD-refeeding groups (*F*_3,18_ = 33.29, *P* = 0.001).

Under the food hoarding condition, no group differences were found in baseline levels of food hoarding (Fig. 2D). RH2h males increased their food hoarding by 1.4 fold 2 hr after food was provided, compared to the control (*t*_12_ = 3.44, *P* = 0.05 for one-tail test; Fig. 2D). Further, the control and RH2h males did not differ in food intake during 2 hr food hoarding (Fig. 2E). There were no significant differences in food hoarding during the experimental course in the control group (*F*_3,18_ = 0.71, *P* = 0.56). However, in the FD-rehoarding group, food hoarding within first 2 hr was much higher than the baseline (*F*_3,18_ = 3.81, *P* = 0.03).

### Neuronal activation associated with food-deprivation and re-feeding

**Figure 3:**
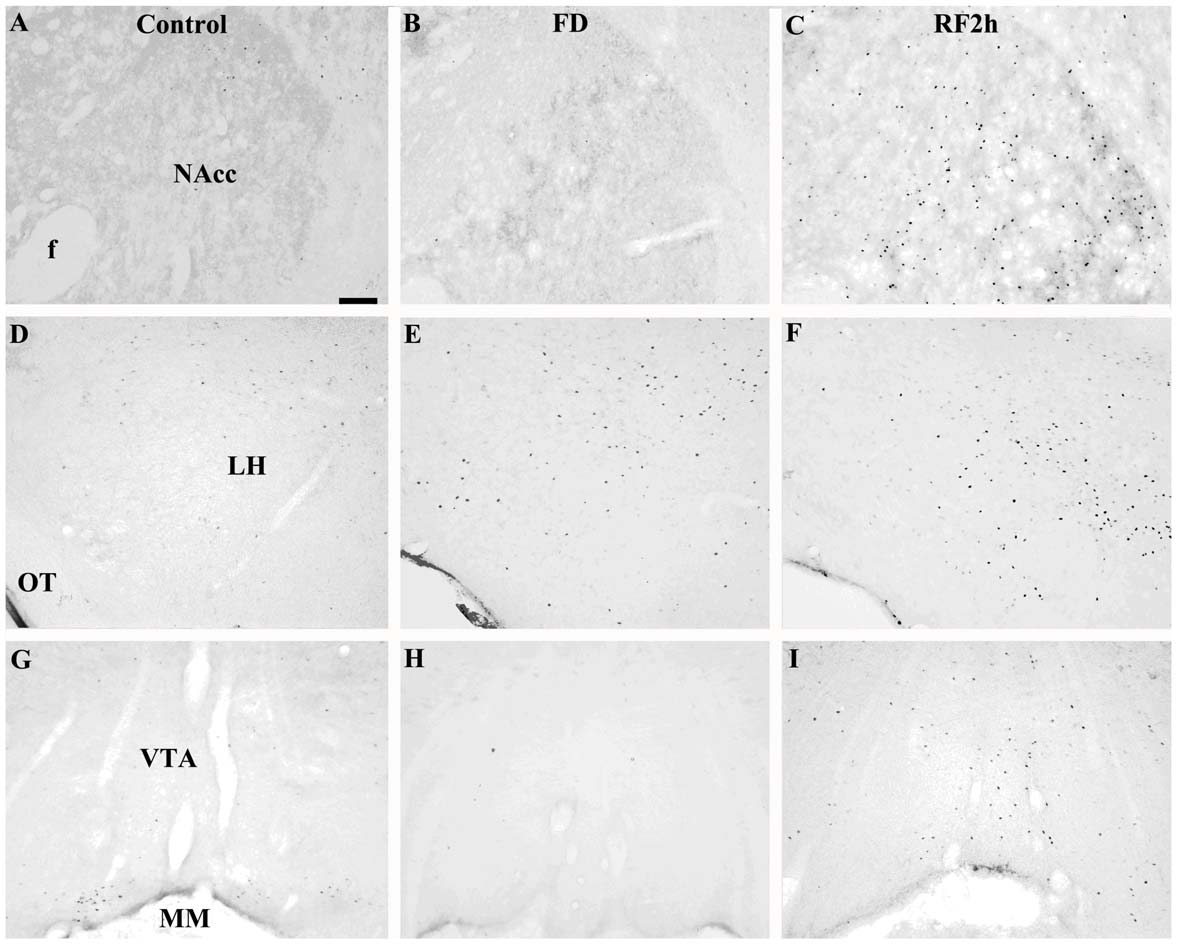
Photomicrographs of Fos-ir cells. Fos-ir cells were shown in the nucleus accumbens (NAcc; panel A–C), lateral hypothalamus (LH; panel D–F), and ventral tegmental area (VTA; panel G–I) in the control (A, D, G), FD (B, E, H), and re-fed voles (RF2h: C, F, I). f, fornix; OT, optic tract; MM, medial mammillary nucleus. Scale bar = 100 µm in panel A (applies to A–I).

Fos-ir was used to indicate neuronal activation following food deprivation and re-feeding in male voles. Under the re-feeding condition, RF2h males showed more Fos-ir labeled cells in many of the brain areas examined than the control and FD males (Table 1). However, no group differences were found in the number of Fos-ir cells in the CeA and ARC. Under the food hoarding condition, control and RH2h males also differed in the number of Fos-ir cells in many brain areas examined (Table 2). It is important to note that in the NAcc and VTA – areas involved in mesolimbic dopamine activity – and in the LH – an area involved in orexin function, significant increases in neuronal activation over baseline were found associated with re-feeding or food hoarding (Fig. 3).

**Table 1:** Fos-ir expression in the selected brain areas of male Brandt's voles that were intact (Control), food-deprived for 48 hr (FD), or re-feeding for 2 hr (RF2h) following FD.

Area	Control	FD	RF2h	*F*	*P*
NAcc	100.0±17.8^b^	56.4±10.8^b^	324.9±40.4^a^	31.48	0.001
LS	100.0±28.3^b^	194.3±38.9^b^	382.3±56.8^a^	10.24	0.001
BNST	100.0±20.4^b^	93.4±17.2^b^	254.4±35.2^a^	12.95	0.001
MPOA	100.0±14.2^b^	103.6±6.3^b^	136.6±8.1^a^	4.52	0.02
ACo	100.0±13.1^b^	124.6±15.7^ab^	159.6±11.2^a^	4.36	0.03
MeA	100.0±12.8^b^	95.5±10.9^b^	183.3±10.2^a^	19.28	0.001
CeA	100.0±13.20	105.0±10.2	128.6±10.3	1.83	0.18
PVT	100.0±12.1^b^	66.4±8.7^b^	331.2±66.6^a^	13.63	0.001
PVN	100.0±29.2^b^	55.0±10.1^b^	245.6±33.8^a^	16.14	0.001
SCN	100.0±18.6^b^	116.4±12.4^b^	266.5±26.4^a^	21.58	0.001
AH	100.0±9.8^b^	108.5±12.0^b^	182.1±26.0^a^	6.48	0.006
DMH	100.0±25.8^b^	105.9±12.9^b^	244.6±22.4^a^	16.50	0.001
ARC	100.0±10.90	156.9±44.3	228.5±96.8	0.98	0.39
LH	100.0±16.7^b^	153.9±18.5^b^	338.1±38.3^a^	21.38	0.001
VTA	100.0±11.0^b^	65.9±9.4^b^	318.1±48.4^a^	22.41	0.001

Data are presented as percentage changes over the control by mean ± SEM. Group differences are expressed as *P*<0.05, and different superscript letters indicate group differences in the number of Fos-ir cells in each brain area.

**Table 2:** Fos-ir expression in the selected brain areas of male Brandt's voles that were intact (Control) or food-deprived for 48 hr followed by 2 hr food hoarding (RH2h).

Area	Control	RH2h	*t*	*P*
NAcc	100.0±25.3	896.4±210.5^**^	3.76	0.003
LS	100.0±34.8	362.0±108.8^*^	2.29	0.04
BNST	100.0±17.1	398.2±56.9^***^	5.02	0.001
MPOA	100.0±23.6	519.8±169.6^*^	2.45	0.03
ACo	100.0±19.1	332.9±44.8^***^	4.79	0.001
MeA	100.0±14.3	218.2±63.0	1.83	0.09
CeA	100.0±51.3	730.9±200.1^**^	3.06	0.01
PVT	100.0±22.8	363.6±62.7^**^	3.95	0.002
PVN	100.0±11.5	364.2±66.9^**^	3.89	0.002
SCN	100.0±25.8	166.4±17.5^*^	2.13	0.05
AH	100.0±27.1	363.8±109.2^*^	2.34	0.04
DMH	100.0±26.8	700.0±148.8^**^	3.97	0.002
ARC	100.0±42.7	416.3±66.4^**^	4.01	0.002
LH	100.0±16.0	329.4±53.1^***^	4.23	0.001
VTA	100.0±48.6	663.4±127.1^***^	4.14	0.001

Data are presented as percentage changes over the control by mean ± SEM. Significant group differences are expressed as * *P*<0.05, ** *P*<0.01, and *** *P*<0.001.

### Activation of TH-ir cells

**Figure 4:**
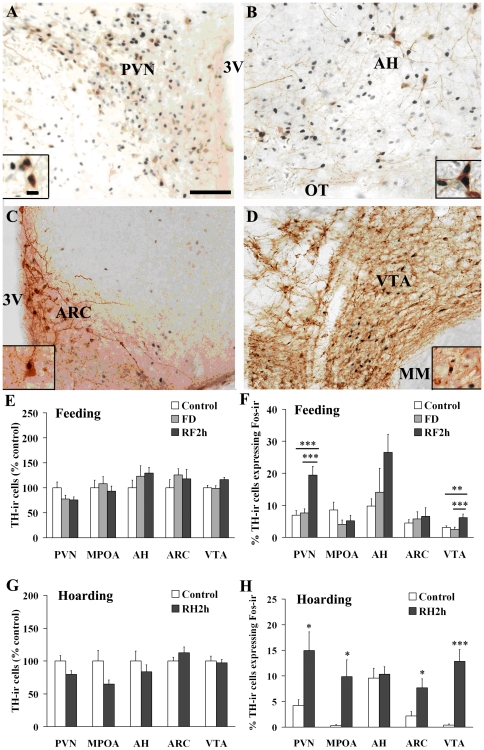
Double-labeled cells for TH-ir and Fos-ir staining. Photomicrographs of double-labeled cells for TH-ir (brown cytoplasmic staining) and Fos-ir (dark nuclear staining) were shown in the paraventricular nucleus (PVN; panel A), anterior hypothalamus (AH; panel B), arcuate nucleus (ARC; panel C), and ventral tegmental area (VTA; panel D) in the brain of male Brandt's voles. 3 V, third ventricle; OT, optic tract; MM, medial mammillary nucleus. Scale bar = 100 µm in panel A (applies to A–D); 10 µm in inset to panel A (applies to insets to A–D). Panel E: No group differences were found in the number of TH-ir cells in any brain areas examined in male voles that were intact controls, food-deprived for 48 hr (FD), or re-fed for 2 hr following FD (RF2h). Panel F: RF2h males had a higher percentage of TH-ir cells that co-labeled for Fos-ir in the PVN and VTA than did the control and FD males. Panel G: Under the food hoarding condition, control males and males that experienced 2 hr food hoarding following FD (RH2h) showed no differences in the number of TH-ir cells in any of the brain regions examined. Panel H: RH2h males, however, had a higher percentage of TH-ir cells co-labeled for Fos-ir in the PVN, MPOA (medial preoptic area), ARC, and VTA than control males. No difference was found in the AH. Data are presented as mean ± SEM. * *P*<0.05, ** *P*<0.01, and *** *P*<0.001.

TH-ir stained cells (brown cytoplasmic staining) were found in many brain areas including the MPOA, AH, PVN, ARC, and VTA (Fig. 4A–D). Many TH-ir cells were also co-labeled with Fos-ir, indicating their activation. Under the re-feeding condition, no differences were found in the number of TH-ir cells in any of the brain areas examined among the control, FD, and RF2h males (Fig. 4E). However, RF2h males showed an increase in the percentage of TH-ir cells that co-expressed Fos-ir in the PVN (*F*_2,21_ = 13.76, *P* = 0.001) and VTA (*F*_2,21_ = 5.91, *P* = 0.01), but not in the MPOA, AH, and ARC, compared to the control or FD males (Fig. 4F). Under the food hoarding condition, no group differences were found in the number of TH-ir cells in the above-mentioned brain areas (Fig. 4G). However, RH2h males had a higher percentage of TH-ir neurons that co-expressed Fos-ir in the MPOA (*t*_12_ = 2.86, *P* = 0.01), PVN (*t*_12_ = 2.81, *P* = 0.02), ARC (*t*_12_ = 2.84, *P* = 0.02), and VTA (*t*_12_ = 5.36, *P* = 0.001), but not in the AH, than did the control males (Fig. 4H).

### Activation of orexin-ir cells in the LH

**Figure 5:**
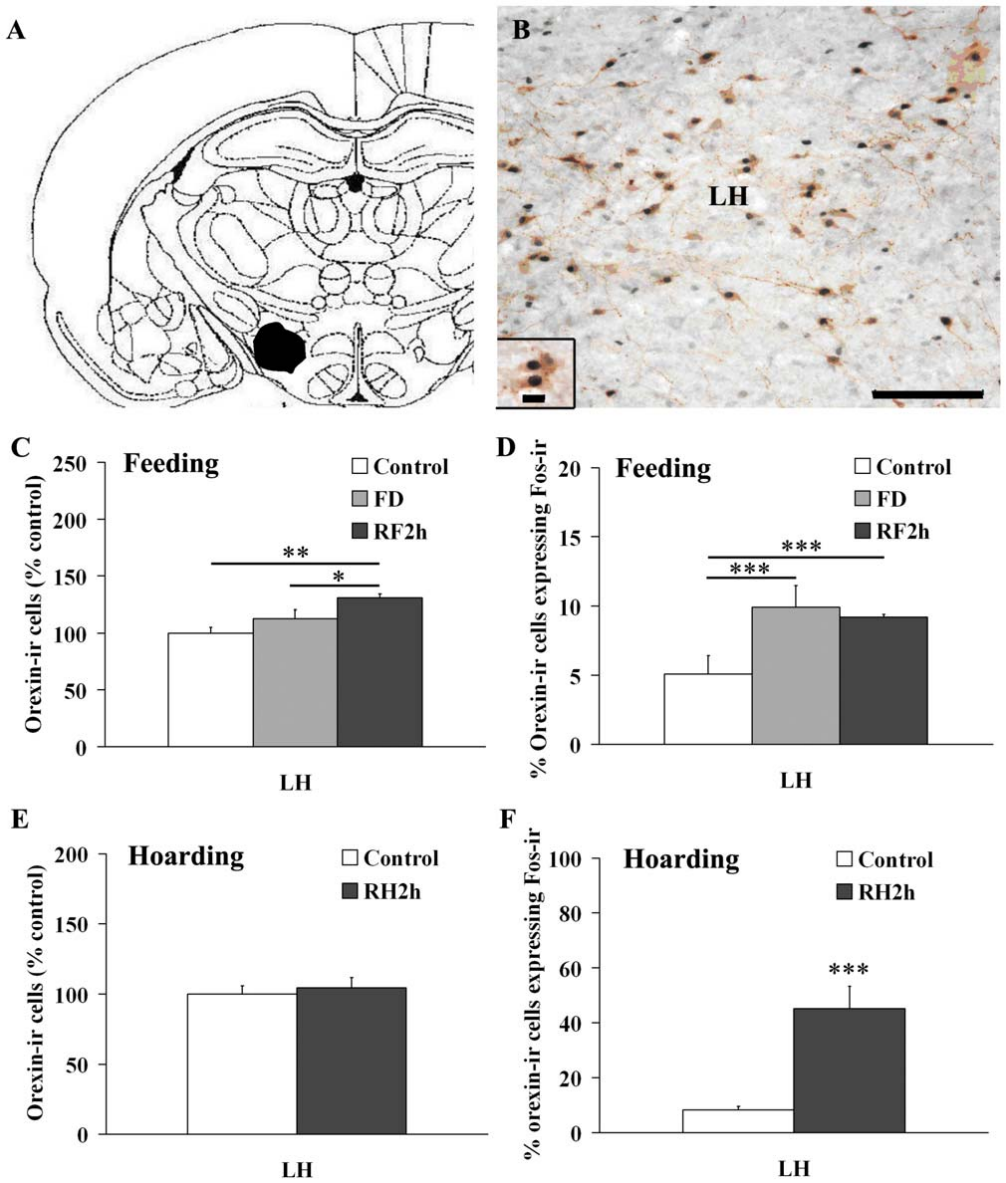
Double-labeled cells for orexin-ir and Fos-ir staining. Schematic drawing (panel A) and photomicrograph (panel B) illustrating the lateral hypothalamus (LH) and cells double-labeled for orexin-ir (brown cytoplasmic staining) and Fos-ir staining in LH in the vole brain. Scale bar = 100 µm in panel B; 10 µm in inset to panel B. Panel C: The males that were re-fed for 2 hr following FD (RF2h) had more orexin-ir cells in the LH than control and food-deprived for 48 hr (FD). Panel D: Both FD and RF2h males had a higher percentage of orexin-ir cells that co-expressed Fos-ir in the LH than the control males. Panel E: Under the food hoarding condition, control males and males that experienced 2 hr food hoarding following FD (RH2h) showed no differences in the number of orexin-ir cells in the LH. Panel F: RH2h males, however, had a higher percentage of orexin-ir cells co-labeled for Fos-ir in the LH than control males. Data are presented as mean ± SEM. * *P*<0.05, ** *P*<0.01, and *** *P*<0.001.

Orexin-ir cells (brown cytoplasmic staining) were found in the LH (Fig. 5A–B). Some orexin-ir cells also co-expressed Fos-ir staining. Under the re-feeding condition, RF2h males had more orexin-ir cells in the LH than control and FD males (*F*_2,21_ = 6.08, *P* = 0.008). Interestingly, the percentage of orexin-ir cells that co-expressed Fos-ir was significantly higher in both FD and RF2h males than in control males (*F*_2,21_ = 4.26, *P* = 0.03; Fig. 5D). Further, under the food hoarding condition, there was no group difference in the number of orexin-ir cells (Fig 5E). However, RH2h males showed a significantly higher percentage of orexin-ir cells co-labeled for Fos-ir in the LH than the control males (*t*_12_ = 4.92, *P* = 0.001; Fig. 5F).

## Discussion

An increase in feeding or food hoarding behavior after a period of food shortage is a species-specific adaptive strategy to restore energy balance and to ensure animal's survival and reproductive success [1]. In the present study, we found that food-deprived male Brandt's voles increased feeding or food hoarding after re-feeding and this effect was dependent upon environmental setting. Interestingly, both feeding and food hoarding induced an increase in neuronal activation in a large number of brain areas examined, including the NAcc, LH and VTA. In addition, feeding and food hoarding significantly increased TH-ir/Fos-ir staining in the VTA and orexin-ir/Fos-ir labeling in the LH. Together, these data indicate that activities of the mesolimbic dopamine and LH orexin systems are associated with feeding and food hoarding, and thus may be involved in mediating enhanced feeding or food hoarding following food deprivation in male Brandt's voles.

### Physiological and behavioral changes following food deprivation and re-feeding

A decline in food availability usually results in a decrease in body weight in many rodent species, such as rats, mice, and hamsters [7]. How an animal copes with body weight loss is of critical importance for its survival in the field. In the present study, a small decrease in body weight and RMR was found in food-deprived male voles. Although not examined in the present study, a previous study reported that Brandt's voles displayed caecotrophy behavior – a common behavior in herbivorous small mammals that involves ingestion of soft faeces derived from caecal contents – which contributed to about 9.1% of total protein intake and 8.2% of the daily intake of metabolizable energy when they were fed on commercial rabbit pellets [31]. Therefore, such caecotrophy behavior may supply extra energy and protect the voles from losing too much body weight under food deprivation. The voles regained body weight and RMR once they had re-access to food shortly after the 48-hr food deprivation period.

Adipocyte-derived leptin levels decreased with food deprivation or restriction [11], [14] and continued to decrease throughout re-feeding for one week in Brandt's voles [15], which may act as a starvation signal to drive increased food intake to compensate for lost energy [32]. Some species display increased feeding in response to food deprivation [8], [33]. For example, jirds (*Meriones shawi*), a desert rodent species, are prodigious hoarders in the field. This species abolished food hoarding behavior and increased feeding following food deprivation [34]. Others species, however, do not show compensatory hyperphagia, despite a rapid fall in plasma leptin. For instance, Syrian and Siberian hamsters displayed a dramatic fasting-induced increase in food hoarding behavior with little or no changes in food intake [9]–[10]. In the present study, food-deprived voles with no opportunities for food hoarding significantly increased their food intake during the first two hours of re-feeding. In contrast, when permitted to hoard food, they did not increase their feeding, but instead, significantly increased food hoarding. Increased food hoarding may enhance an animal's competitive status for limited resources and ensure a continuous supply of energy, while increasing feeding may just satisfy a short-time demand for energy [35]. Our data suggest that male Brandt's voles display different behavioral strategies dependent upon environmental setting, and they have evolved to preferentially increase food hoarding rather than feeding in response to energetic challenges.

### Neuronal activation associated with re-feeding following food deprivation

Previous studies in rats have shown that the hypothalamic nuclei including the PVN, DMH, LH, and ARC are involved in food-entrained homeostatic regulation [36]–[39]. The PVN, DMH and LH receive projections from the ARC which integrates peripheral metabolic signals, such as leptin, and sends efferent signals to regulate energy homeostasis and reproduction [12], [14]. In the present study, we found activation of the above-mentioned nuclei by feeding or food hoarding following food deprivation. We also found that some forebrain regions involved in the stress response, including the NAcc, LS, BNST, amygdala and VTA, were activated by feeding or food hoarding in food deprived male voles. It has been shown that food restriction induced increases in arousal in mice [40], depression- and anxiety-like behaviors in rats [41], and improved spatial memory in rats [42]. A palatable meal or re-feeding after food deprivation could enhance locomotor activity, and motivated behavior for drugs of abuse as well as for food in rats [43]–[44]. All of these neural and behavioral data suggest that forebrain circuits involved in arousal, motivation and reward may be involved in mediating FD-induced increases in feeding and hoarding behaviors.

### Dopamine and orexin involvement in re-feeding

Activation of the VTA-NAcc reward pathway may promote food consumption and food hoarding [45]–[46]. In rats, dopamine turnover in the NAcc was increased by feeding [47], and blocking dopamine D2 receptors curtailed the beneficial impact of calorie restriction on glucose tolerance of high-fat diet induced obese mice [48], implicating an involvement of this dopamine system in metabolic process. Our previous data showed that increased food hoarding in male Mongolian gerbils (*Meriones unguiculatus*) was associated with activation of VTA dopamine neurons [27]. The present study provided further evidence in another species to support that dopamine neurons in the VTA were markedly activated by feeding and food hoarding following food deprivation. It has been shown that food deprivation, by lowering circulating leptin levels, strongly augmented the motivation for addictive drugs and palatable foods [46], [49]–[50]. A long-term RNAi-mediated knockdown of leptin receptors in the VTA led to increased food intake and sensitivity to highly palatable food, whereas administration of leptin to the VTA resulted in reduced food intake [51]. These data suggest that the activation of the dopamine system which may be mediated by leptin signal may facilitate selection of the appropriate behavioral response - feeding vs hoarding - following food deprivation.

In the present study, feeding and food hoarding following food deprivation also significantly activated orexin-ir neurons in the LH. Further, re-feeding was found to be associated with an increase in the number of orexin-ir neurons in the LH, whereas food deprivation alone was sufficient to increase the number of orexin-ir/Fos-ir double-labeled cells in the LH. Therefore, the LH orexin system may respond to the stimuli associated with food deprivation and/or subsequent feeding and food hoarding by increasing the number of cells that expressed orexin as well as by activating more orexin expressing neurons in the LH. These data provide further evidence to support the notion that orexin neurons are involved in cognitive and behavioral responses to highly arousing conditions including both aversive (e.g., anxiety and depression associated with food deprivation) and appetitive (e.g., motivation for food during re-feeding) stimuli [20], [52]. Leptin receptors are located on orexin-containing neurons in the LH [53], indicating that the hypothalamic orexin system may respond to metabolic signals and regulate feeding behavior [54]. Indeed, orexin neurons in the LH were also activated in response to hypoglycemia [55] and food restriction in rats and mice [56]–[58]. Moreover, central administration of orexin stimulated food intake and locomotor activity in rats [59]–[60], whereas genetic or pharmacological ablation of orexin neurons reduced anticipatory locomotor activity induced by re-feeding in mice [56], and attenuated reward-based feeding of palatable foods in rats [61].

In Brandt's voles, similar changes in activity of the dopamine and orexin systems in response to feeding and food hoarding may indicate interactions between the two neurochemical systems in the regulation of these behaviors. This notion is supported by some existing data. For example, orexin receptors are widely distributed not only in the hypothalamus [62] and PVT [63], but also in the NAcc [64] and VTA [65], wherein the mesolimbic dopamine system is localized. Microinjections of orexin into the VTA activated dopamine neurons, facilitated dopamine production [66], reinstated cocaine seeking [67], and increased intake of palatable foods [68]. On the other hand, orexin neurons in the LH are embedded with a dense plexus of dopamine fibers [69]. Administration of a dopamine receptor agonist into the NAcc increased Fos expression in orexin neurons in the LH in rats [70]. Other studies have also shown that orexin neurons and fibers may mediate the aversive effects of morphine withdrawal [20]–[21], [71]. Therefore, central dopamine and orexin systems may interact to integrate environmental cues and to participate in a variety of behavioral and physiological processes associated with high-arousal environmental conditions. Needless to say, the causal relationship between dopamine/orexin and feeding/food hoarding needs to be further examined in pharmacological studies.

In summary, we have shown that male Brandt's voles displayed an increase in food hoarding (appetitive behavior) or feeding (consummatory behavior) [17], and preferred the former behavior over the latter in response to energetic challenges when they had options in a lab setting environment. Our data also indicated that mesolimbic dopamine and LH orexin neurons were activated shortly after re-feeding or food hoarding following food deprivation. Therefore, coordinated activations of central dopamine and orexin systems may play an important role in mediating increased feeding/hoarding behavior induced by food deprivation. This, in turn, may serve an essential evolutionary adaptation for animal's survival and reproductive success.
